# Felid Parasitoses, New Insights and Open Perspectives

**DOI:** 10.3390/pathogens11010028

**Published:** 2021-12-28

**Authors:** Anastasia Diakou, Simone Morelli, Donato Traversa

**Affiliations:** 1 Faculty of Health Sciences, School of Veterinary Medicine, Aristotle University of Thessaloniki, 54124 Thessaloniki, Greece; diakou@vet.auth.gr; 2 Faculty of Veterinary Medicine, University of Teramo, 64100 Teramo, Italy; smorelli@unite.it

## 1. Introduction

Felids are fascinating animals. From the large wild felids of the jungles and savannas to the domestic cat, which according to Victor Hugo “was created by God to give humans the pleasure of petting a tiger”, felids generate feelings of wonder, admiration and awe. The ecology, biology, behavior, and pathophysiology of these animals are still under-investigated fields, and erroneous application of facts that are valid for other animal families (e.g., canids) to felids is a common trap that should be avoided [1]. In the field of Parasitology, a steadily increasing number of surveys have elevated the importance of exploring felid parasites and parasitic diseases in recent decades. In an effort to highlight the ongoing research, the new data collected, and the accumulating knowledge on important felid parasitoses, this Special Issue entitled “Felid Parasitoses, New Insights and Open Perspectives” was established. This editorial summarizes the data reported in this Special Issue, consisting of thirteen papers: 11 research articles and 2 reviews. These contributions provide valuable results from epizootiological studies, first case reports, advances in diagnostic tools and insights accumulated by the intense efforts in felid parasitology research over recent years.

## 2. Felid Cardiopulmonary Parasites—Expanding Knowledge

Six papers of this Special Issue are dedicated to felid cardiopulmonary parasites. *Aelurostrongylus abstrusus* is the most common feline lungworm all over the globe. However, there are still areas of the world where epizootiological data are poor or non-existent. In the survey conducted by Lopez-Osorio et al. [2], the presence of aelurostrongylosis in domestic cats from Antioquia, Colombia, was reported for the first time. The confirmation of *A. abstrusus* occurrence in this area, albeit at a relatively low prevalence (0.4%), contributes to overall knowledge about its geographical distribution, and updates the differential diagnosis list that should be taken into consideration at a clinical level. Brazil is also a country with poor epizootiological data regarding *A. abstrusus* in cats. The results reported by da Silva Lima et al. [3] represent the first depiction of prevalence for this important pathogen in cats living in the Brazilian Amazon. Furthermore, this survey reports the first molecular confirmation of this parasite in Brazil and describes the clinical picture of one of the cases detected among the animals examined [3]. 

Even in areas where feline aelurostrongylosis is a known enzootic disease, the factual prevalence of *A. abstrusus* infection may be, in some cases, underestimated. Serodiagnosis—although giving a positive result several weeks after successful treatment and, thus, not always being related to an active infection—is of great diagnostic value because it may detect anti-*A. abstrusus* antibodies before patency; this technique can also be useful in cases with negative fecal examination results due to intermittent or low larval shedding, which represents an important diagnostic challenge. Thus, serodiagnosis uncovers the actual risk of exposure of a cat population to the parasite. Accordingly, Schnyder et al. [4] examined a large number of cat sera samples from all over Germany by a validated ELISA, and found that 12.0% of the animals were seropositive. This is a significantly higher prevalence than those determined by coprological examinations performed in the country in the past. 

Indeed, diagnosis of aelurostrongylosis in cats is often challenging. In their comprehensive study, Raue et al. [5] investigated the evolution of experimental *A. abstrusus* infection in cats using fecal examination (the Baermann method), PCR, serology, and imaging techniques; they concluded that a multi-diagnostic approach is often necessary and should be considered both in clinical settings and in modern anthelmintic efficacy studies in order to avoid post mortem evaluations.

Other than *A. abstrusus*, major felid cardiopulmonary nematodes include the species *Troglostrongylus brevior*, *Capillaria aerophila*, *Angiostrongylus chabaudi* (mainly in wildcats) and some rarely reported species, i.e., *Oslerus rostratus* and *Troglostongylus subcrenatus*. During the last decade, these nematodes have been intensively studied. A lot of questions have been asked and light has been shed on the least researched areas of felid cardiopulmonary parasites. This large amount of research is presented and analyzed critically in the two review articles of this Special Issue [6,7]. Traversa et al. [6] take up the thread from the start of the felid cardiopulmonary parasites research boom in early 2010, and navigate through major issues, i.e., parasite identification dilemmas, hosts/parasites ecology, new data about transmission, epizootiology, new questions spawned, and the new research perspectives created. From an applied parasitology point of view, Morelli et al. [7] summarized the current knowledge on the clinical presentation, imaging findings, diagnostic tools, and treatment/prevention options for these important feline pathogens, providing all necessary information for timely and accurate diagnosis, clinical management, and control of feline cardiopulmonary parasites.

## 3. Parasites in Wild Felids—Valuable Information about These Key Players

Information about the parasites of wild felids is scant, mainly because of the inherent challenges in collecting biological material from these animals, especially from natural environments. Thus, every report providing solid data about these parasites is extremely valuable to the field. This Special Issue includes three papers on parasites in wild felids. Diakou et al. [8] investigated the endoparasite fauna of European wildcats in Greece. Both copromicroscopical and post mortem examinations were included in this survey, providing a comprehensive picture of the parasites and their frequency in wildcats living in the area. Furthermore, new host records and new distribution data for certain parasites are reported. Informative photographs and analyses of the epizootiology, clinical impact and zoonotic potential of the parasites found are also presented in this paper [8]. 

In Spain, the endoparasites of the reintroduced Iberian lynx and some sympatric wild mesocarnivores were investigated by copromicroscopical examinations by Figueiredo et al. [9]. As the authors suggest, it is important to maintain surveillance in reintroduction programs of endangered species, and monitoring of pathogens must include sympatric wild animal species as well as domestic animals that may share the same parasites, so that preventive measures can be applied in a timely manner [9]. Endoparasites found in wild felids (jaguar, ocelot, puma, and jaguarundi) from Colombia are reported by Uribe et al. [10]. The results of this study are based on copromicroscopical, post mortem, and molecular (PCR) examinations and demonstrate the potential role of wild felids as natural reservoir hosts for important zoonotic parasites, highlighting the necessity of management practices in conservation strategies promoting animal and human health.

## 4. New Insights in Classical and Lesser Studied Feline Parasites

*Toxoplasma gondii* and *Otodectes cynotis* could be considered well known, classical concerns in felid parasitology that remain highly relevant due to their clinical and, regarding *T. gondi*, economic and zoonotic importance. Attipa et al. [11] investigated the seroprevalence of *T. gondi* infection in cats from Cyprus for the first time. According to the results, 32.3% of the examined cats were seropositive. Feline immunodeficiency virus seropositivity and a lack of any vaccination were associated with *T. gondii* seropositivity in a statistically significant manner [11]. Serodiagnosis of *T. gondii* in stray cats may be challenging due to difficulties with sample collection. Simon et al. [12] validated the use of dried blood samples for the detection of *T. gondii* antibodies in stray cats, as an easy and cheap alternative to classical blood collection and serum separation before examination. The results indicate that applying the modified agglutination test to blood dried on blotting paper is reliable for identifying the presence of *T. gondii* antibodies in domestic cats, offering a practical technique for monitoring free-ranging animals [12].

The cosmopolitan mite *O. cynotis* is the primary agent of otitis externa in cats. Ribeiro Campos et al. [13] evaluated the efficacy of the ectoparasiticide drug sarolaner, administrated orally, for the treatment of *O. cynotis* infestation in cats for the first time. This treatment produced satisfactory results with no adverse effects, so it is suggested as a good option for the resolution of otodectic mange in cats, offering some safety advantages over topical products. 

In contrast to the abovementioned, relatively well-known feline parasites, the metastrongyloid nematode *Gurltia paralysans* is still a mystery in many aspects. Its life cycle is not known, although it is presumably heteroxenous. It seems to be geographically limited to South America, but it has also recently been found in Tenerife Island, Spain, even though the case was not confirmed as autochthonous. An important difficulty in investigating the occurrence of this parasite is the fact that the diagnostic stage is unknown and diagnosis to date is only achieved in post mortem examination. In their study, Gómez et al. [14] evaluated a commercial serological test, designed for the diagnosis of canine angiostrongylosis due to *Angiostrongylus vasorum*, for an intra vitam diagnosis of *G. paralysans* infection in cats. According to the results, a cross-reaction between *A. vasorum*-specific antigens and *G. paralysans* offers the possibility of using this antigen test as a diagnostic method for feline gurltiosis in live domestic cats. Furthermore, this study presents the clinical and histopathological findings of four cases of feline natural infections, providing important insights on this neglected and little-known feline parasite [14].

## 5. Conclusions

Felid parasitology is a dynamic field of continuous development and expansion. This Special Issue entitled “Felid Parasitoses, New Insights and Open Perspectives” was an opportunity for both experienced and young scientists to present their work, share their findings and inquiries, and ultimately encourage and inspire further research and discoveries in this exciting and rewarding area of science.
